# Potential coupling of microbial methane, nitrogen, and sulphur cycling in the Okinawa Trough cold seep sediments

**DOI:** 10.1128/spectrum.03490-23

**Published:** 2024-05-01

**Authors:** Ye Chen, Xiyang Dong, Zhilei Sun, Cuiling Xu, Xilin Zhang, Shuangshuang Qin, Wei Geng, Hong Cao, Bin Zhai, Xuecheng Li, Nengyou Wu

**Affiliations:** 1 Key Laboratory of Gas Hydrate, Qingdao Institute of Marine Geology, Ministry of Natural Resources, Qingdao, China; 2 Laboratory for Marine Mineral Resources, Qingdao Marine Science and Technology Center, Qingdao, China; 3 Key Laboratory of Marine Genetic Resources, Third Institute of Oceanography, Ministry of Natural Resources, Xiamen, China; 4 Key Laboratory of Marine Chemistry Theory and Technology, Ministry of Education, College of Chemistry and Chemical Engineering, Ocean University of China, Qingdao, China; 5 China Offshore Fugro Geosolutions (Shenzhen)Co.Ltd., Shenzhen, China; University of Minnesota, St. Paul, Minnesota, USA

**Keywords:** metagenomics, microbial nutrient cycling, potential coupling, cold seep sediments, Okinawa Trough

## Abstract

The Okinawa Trough (OT) is a back-arc basin with a wide distribution of active cold seep systems. However, our understanding of the metabolic function of microbial communities in the cold seep sediments of the OT remains limited. In this study, we investigated the vertical profiles of functional genes involved in methane, nitrogen, and sulphur cycling in the cold seep sediments of the OT. Furthermore, we explored the possible coupling mechanisms between these biogeochemical cycles. The study revealed that the majority of genes associated with the nitrogen and sulphur cycles were most abundant in the surface sediment layers. However, only the key genes responsible for sulphur disproportionation (*sor*), nitrogen fixation (*nifDKH*), and methane metabolism (*mcrABG*) were more prevalent within sulfate-methane transition zone (SMTZ). Significant positive correlations (*P* < 0.05) were observed between functional genes involved in sulphur oxidation, thiosulphate disproportionation with denitrification, and dissimilatory nitrate reduction to ammonium (DNRA), as well as between AOM/methanogenesis and nitrogen fixation, and between sulphur disproportionation and AOM. A genome of *Filomicrobium* (class Alphaproteobacteria) has demonstrated potential in chemoautotrophic activities, particularly in coupling DNRA and denitrification with sulphur oxidation. Additionally, the characterized sulfate reducers such as Syntrophobacterales have been found to be capable of utilizing nitrate as an electron acceptor. The predominant methanogenic/methanotrophic groups in the OT sediments were identified as H_2_-dependent methylotrophic methanogens (Methanomassiliicoccales and Methanofastidiosales) and ANME-1a. This study offered a thorough understanding of microbial ecosystems in the OT cold seep sediments, emphasizing their contribution to nutrient cycling.

**IMPORTANCE:**

The Okinawa Trough (OT) is a back-arc basin formed by extension within the continental lithosphere behind the Ryukyu Trench arc system. Cold seeps are widespread in the OT. While some studies have explored microbial communities in OT cold seep sediments, their metabolic potential remains largely unknown. In this study, we used metagenomic analysis to enhance comprehension of the microbial community's role in nutrient cycling and proposed hypotheses on the coupling process and mechanisms involved in biogeochemical cycles. It was revealed that multiple metabolic pathways can be performed by a single organism or microbes that interact with each other to carry out various biogeochemical cycling. This data set provided a genomic road map on microbial nutrient cycling in OT sediment microbial communities.

## INTRODUCTION

Cold seeps are typically found at the edges of continental shelves, where deeply sourced hydrocarbon-rich fluids migrate upwards from the sedimentary column to the seafloor (1). These ecosystems host abundant and diverse microbes that significantly impact biogeochemical cycles. Anaerobic oxidation of methane (AOM) coupled with sulfate reduction (S-AOM) is a critical biogeochemical process in cold seeps, facilitated by a partnership between anaerobic methanotrophic archaea (ANME) and sulfate-reducing bacteria (SRBs) (2, 3). Although methane emissions from cold seeps intensify the greenhouse effect and ocean warming, the AOM process absorbs nearly 88% of this methane before it escapes into the sea on a global scale (4). Furthermore, it has been discovered that the deep-sea cold seep sediments harbor a wide range of diazotrophs that make a significant contribution to the global nitrogen balance (5). In addition, high rates of nitrogen removal resulting from denitrification have been observed in cold seep sediments from the Gulf of Mexico (6). Consequently, the cold seep is a hotspot for the study of unique life process and biogeochemical cycles.

Microbial interactions are prevalent in the cold seep ecosystem, where they constantly interact with each other to carry out various biogeochemical cycling processes. For instance, research has shown that sulfate-reducing bacteria (SRBs) can form syntrophic relationships with archaeal anaerobic methanotrophs (ANME) through direct electron transfer (7–9) or zerovalent sulphur (10). Additionally, abundant sulphur-oxidizing bacteria (SOB) in the SMTZ may recycle sulfide and produce sulphate to support the syntrophic SRB associated with ANME (11). It is important to note that multiple metabolic pathways can be performed by a single organism. For example, nitrate-reducing sulfide oxidizers can couple sulfide oxidation with nitrate reduction (11). Additionally, various functional microbes, such as methanogens, ANMEs, and SRBs, have been identified as diazotrophs (5). However, due to the high environmental heterogeneity and complex of the cold seep ecosystem, further exploration of the metabolic potential of microorganisms is necessary.

The Okinawa Trough (OT) is a back-arc basin formed by extension within the continental lithosphere behind the Ryukyu Trench arc system. One of its notable geological features is the methane seeps that are linked to gas hydrates(12, 13). Since 0.5 Ma, sedimentation rates have increased up to 0.4 cm yr ^−1^, which is conducive to the preservation and transformation of organic matter (14). Furthermore, mud diapirs, mud volcanoes, and seafloor pockmarks have been identified in the middle and southern sections of the Okinawa, which could sever good migration pathways for free gas migrating upwards (15). Extensive geophysical and geochemical surveys have significantly contributed to the understanding of methane seepage in the OT (16–19). However, only a limited number of studies have examined microbial communities in OT cold seep sediments using high-throughput 16S rRNA gene sequencing (20, 21). The studies found that ANMEs were highly diverse and abundant (20) and that methane seepage intensity significantly impacted the microbial communities in the OT (21). To the best of our knowledge, no study has ever explored the functional role of the microbial community in the OT cold seep sediments.

This study examines the metabolic potential of microorganisms involved in methane, nitrogen, and sulphur cycling, as well as their coupling in OT cold seep sediments. Our study aimed to (i) determine the vertical profile of methane, nitrogen, and sulphur cycling genes and pathways in the cold seep sediments of the OT; (ii) obtain partial and near-complete genomes to reconstruct the metabolic pathways of numerous community members; and (iii) explore the potential coupling between methane, nitrogen, and sulphur cycling. This research has provided a deeper understanding of how the microbial community contributes to nutrient cycling in the OT cold seep sediments.

## MATERIALS AND METHODS

### Sampling site description

The sediment core named G02 was collected in July–August 2020 from the mid-Okinawa Trough area during the R/V “Haiyang Dizhi nine expedition” using a gravity corer (Fig. S1). At this site, obvious methane seepage was tracked using multibeam systems and thriving mussels were observed (20, 22). The gravity core was approximately 145 cm long. The deep-sea cores emitted a noticeable smell of hydrogen sulfide, indicating a predominantly reducing environment. The sediment core consisted of grey-black silty clay, containing noticeable carbonate pebbles and large clam shells in multiple layers. After retrieval, gravity cores were promptly sectioned at 15 cm intervals. Samples for molecular analysis were transported to the laboratory on dry ice and stored at −80°C at laboratory.

The vertical distribution of porewater geochemistry and sedimentary environmental factors in core G02 are displayed in Fig. S2 and have been described in previous studies (20, 23). The total organic carbon (TOC) and total nitrogen (TN) contents in G02 ranged from 0.33% to 1.30% and from 0.1% to 0.18%, respectively, and both decreased with the depth. There was a distinct sulfate-methane transition zone (SMTZ) at depths below 34 cm below the seafloor (cmbsf), characterized by a sharp decrease in sulfate concentration and a drastic increase in methane and hydrogen sulfide concentrations. The isotopic composition of CH_4_ suggests that the gas released in this area is primarily thermogenic in origin, as indicated by its stable C and H isotopic ratios (16). The G02 sample exhibited significantly higher S_pyrite_ contents (65.9–31 μmol/g) compared to acid-volatile sulfide (AVS) (0.24–2.02 μmol/g) and elemental S (S^0^) (0.49–7.94 μmol/g), indicating that sulphur accumulates as S_pyrite_ rather than S^0^ and ASV. Based on the total reduced inorganic sulphur (TRIS = AVS + S^0^+S_py_)-TOC data, intense pyrite formation in the SMTZ was facilitated by SR-AOM (23).

### Metagenome sequencing

DNA was extracted from the sediment samples using the PowerSoil DNA Isolation Kit (12888-100; QIAGEN) according to the manufacturer's instructions. Metagenomic library preparation and DNA sequencing using Illumina Novaseq 6000 were performed at the Center for Qingdao OE Biotech Co., Ltd.

### Read-based taxonomic and functional analyses

Quality control, raw data trimming, and PCR primers removal were performed using Fastp v0.2.3.1 (24). Full-length 16S rRNA genes were retrieved from the metagenome with Phyloflash v3.0 (25) using the SILVA database (release 138.1) for taxonomic annotations. DiTing (26) was used to determine the relative abundances of functional genes related to key biogeochemical cycles, including methane, nitrogen, and sulphur cycles in different samples.

### Metagenome assembly, binning, and genome annotations

Clean reads from the cold seep sediment samples were assembled using MEGAHIT (v1.2.9) (27) with default parameters. Assembled contigs ≥1,000 bp for each metagenome were binned using single-sample and multi-sample binning. For single-sample binning, we used the metaWRAP binning module (parameters: -maxbin2 -concoct -metabat2) (28), Rosella (https://github.com/rhysnewell/rosella), and Semibin (29) to bin each metagenomic assembly. The DAS tool (v. 1.1.4) (30) was used to consolidate the resulting five bin sets for each assembly. For multi-sample binning, we concatenated the individual assemblies from the OT site into a single database and used the VAMB tool (v3.0.2; default parameters) to bin them (31). Bins generated from the multi-sample binning approach were refined using the DAS_Tool (v. 1.1.4). Finally, all resulting bins were combined and dereplicated at a 95% average nucleotide identity (ANI) using dRep (v 3.4.0) (32). The completeness, contamination, and strain heterogeneity of MAGs were evaluated using CheckM (v 1.2.1) (33). Only MAGs with at least 50% completeness and no more than 10% contamination were retained. CoverM v0.4.0 (https://github.com/wwood/CoverM) was used to calculate the relative abundance of MAGs retained at the species level. The taxonomy of each MAG was assigned using GTDB-TK (v 2.1.1) with reference to GTDB R07-RS207 (34). The metabolic reconstruction of each MAG was performed using METABOLIC v4.0 (35), DRAM (36) and FeGenie v2.0 (37).

### Phylogenetic tree construction

Reference genomes accessed from NCBI GenBank and the MAGs from this study were used to construct the phylogenomic tree based on the concatenation of 43 conserved single-copy genes extracted by CheckM (33). Maximum-likelihood trees were constructed using IQ-TREE v2.2.03 and bootstrapped with 1,000 replicates (38). Phylogenetic trees for the *mcrA*, *dsrA*, and *omcZ* genes were generated using MEGA11 for the maximum-likelihood (ML) analysis (https://www.megasoftware.net/) .

## RESULTS

### Microbial diversity characterization

**Fig 1:**
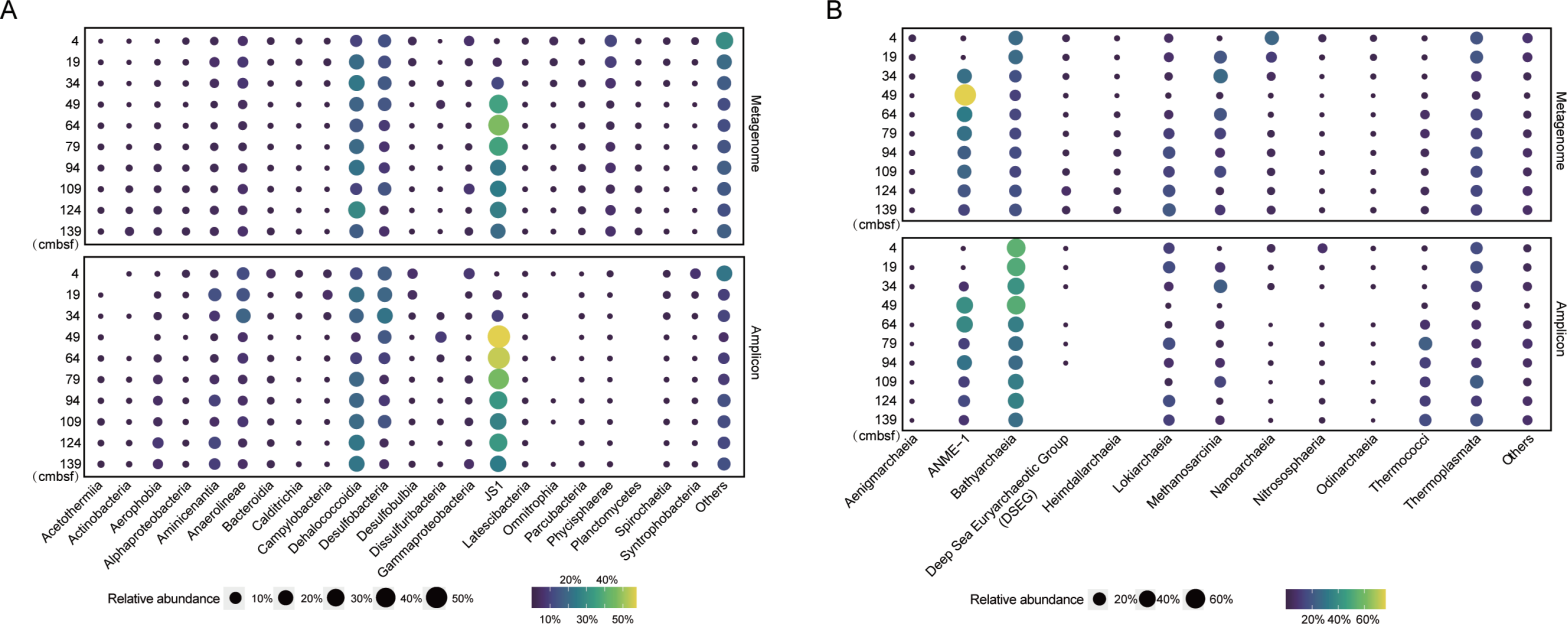
Compositions of microbial communities at different sediment depths based on 16S rRNA genes. Relative abundances of bacterial taxa at different sediment depths (**A**). Relative abundances of archaeal taxa at different sediment depths (**B**). The panels at the top display 16S rRNA gene fragments obtained from metagenomic libraries using the phyloFlash pipeline. The panels at the bottom show 16S rRNA gene amplicons from our previous study (20).

A total of 293,847,376–411,143,146 quality filtered reads were generated from total genomic DNA by the Illumina Novaseq 6000 NextSeq sequencing (Table S1). The phyloFlash pipeline was used for taxonomic profile analysis. The most dominant bacterial lineage in the OT cold seep sediments was JS1, followed by Desulfobacteria, Dehalococcoidia, and Anaerolineae (Fig. 1A). Among them, Desulfobacteria and Anaerolineae were dominant in the above SMTZ (4–34 cmbsf), whereas the JS1 group predominated in the SMTZ (Fig. 1A). The identified archaeal sequences primarily belonged to Bathyarchaeota, ANME-1, Thermoplasmata, Nanoarchaeia, Methanosarcinia, and Lokiarchaeota (Fig. 1B). ANME-1 (mainly ANME-1a) and Lokiarchaeota were more dominant in the SMTZ, whereas Nanoarchaeia were more prevalent in the above sediments of the SMTZ (Fig. 1B). The taxonomic profiles produced by 16S rRNA gene amplicon sequencing, as previously described (20), were comparable to metagenomic profiling (Fig. 1). However, there were differences in the relative abundances of certain groups, such as Dehalococcoidia and Bathyarchaeota (Fig. 1).

Putative methanogens/ANME and sulfate reducing bacteria (SRBs) were recovered. The predominant methanogens in the OT sediments were the H_2_-dependent methylotrophic methanogens Methanomassiliicoccales and Methanofastidiosales (Fig. S3A). The ANME present in the OT cold seep sediments included ANME-1a, ANME-2a-2b, ANME-2c, and ANME-3. ANME-3 was the most abundant in surface sediments but was present in low abundance in subsurface sediments (Fig. S3A). ANME-1a dominated in the SMTZ, which had the highest relative abundance (76% of the archaeal community) at the 49 cmbsf (Fig. S3A). SEEP-SRB1 was the most abundant SRB group, which exhibited a similar trend to the relative abundance of ANME-1a (Fig. S3B and C). In addition, *Desulfatiglans* were the dominant SRB group in the upper sediments (4–49 cmbsf) of the OT cold seep sediments (Fig. S3B).

### Vertical distribution of methane/N/S cycling genes/pathways and their coupling

**Fig 2:**
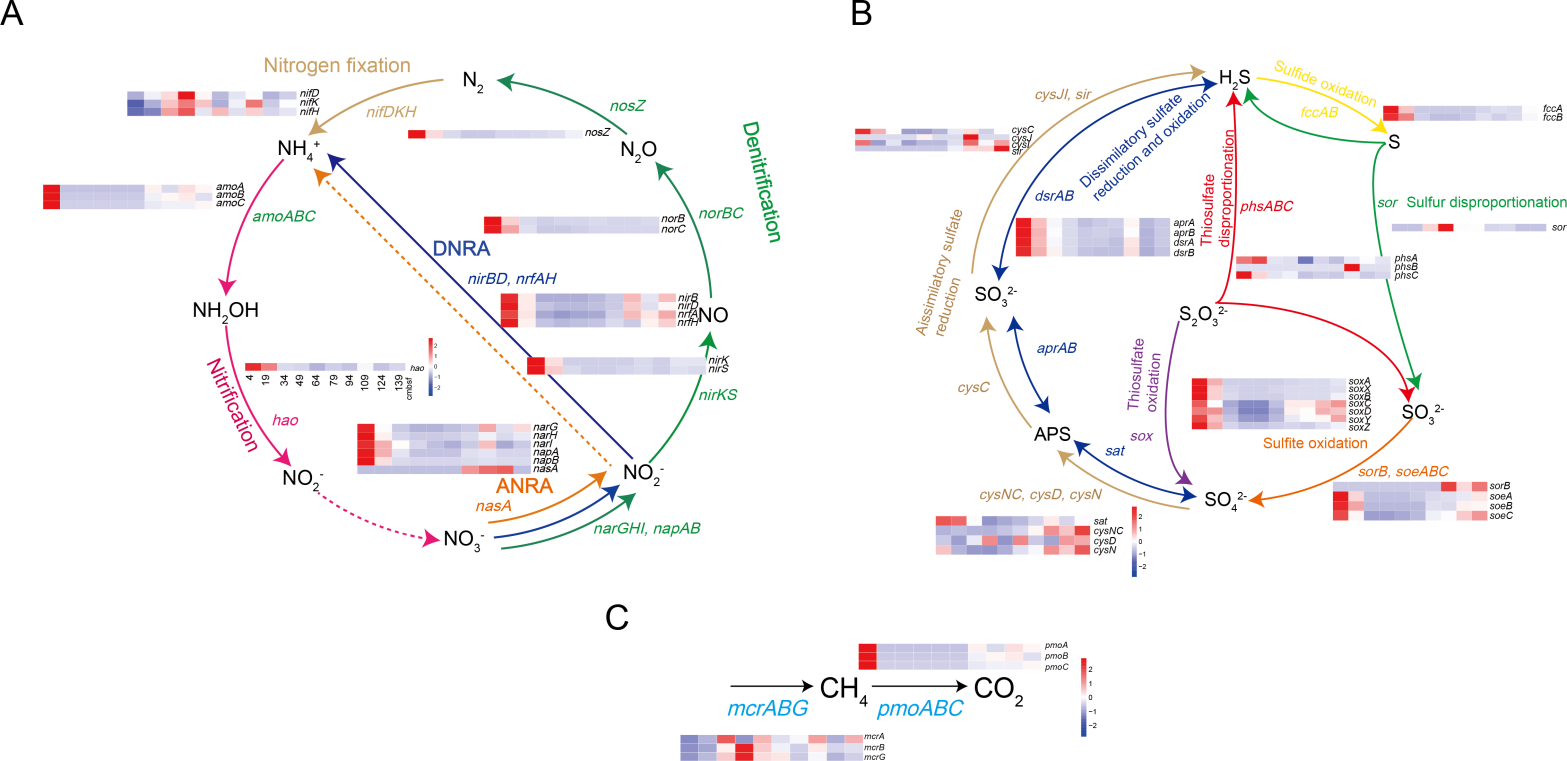
Heatmaps showing the normalized relative abundances (Z-score of, by row) of key genes involved in nitrogen (A), sulphur (B), and methane cycles (C). The dashed line indicates a step without genes.

To explore the potential functions of methane, sulphur, and nitrogen biogeochemical cycles in the OT sediments, we analyzed the depth distribution of different microbial functional genes using DiTing (Fig. 2). The surface sediment showed the highest relative abundance of genes involved in nitrification (*hao*), denitrification (*narGHI*, *napAB*, *nirKS*, *norBC*, and *norZ*), and DNRA (*nirBD* and *nrfAH*). In contrast, the subsurface layers within the SMTZ (below 34 cm) had a higher relative abundance of genes responsible for the nitrogen fixation pathway (*nifDKH*) (Fig. 2A). Regarding the sulphur cycle, genes responsible for dissimilatory sulphate reduction and oxidation (*sat*, *aprAB*, and *dsrAB*), sulfide oxidation (*fccAB*), thiosulfate oxidation (*sox*), sulfite oxidation (*soeABC*), and thiosulfate disproportionation (*phsAC*) were found to be more abundant in the above SMTZ (4 cmbsf and 19 cmbsf) (Fig. 2B). In contrast, the *sor* gene responsible for sulphur disproportionation was highly abundant in the SMTZ sediments at depths of 34–79 cmbsf (Fig. 2B). The marker gene (*mcrA*) involved in both methanogenesis and AOM was mainly enriched in the SMTZ (34–139 cmbsf), whereas the methane oxidation gene (*pmoABC*) showed a higher abundance in the surface sediment layer (4 cmbsf) (Fig. 2C).

**Fig 3:**
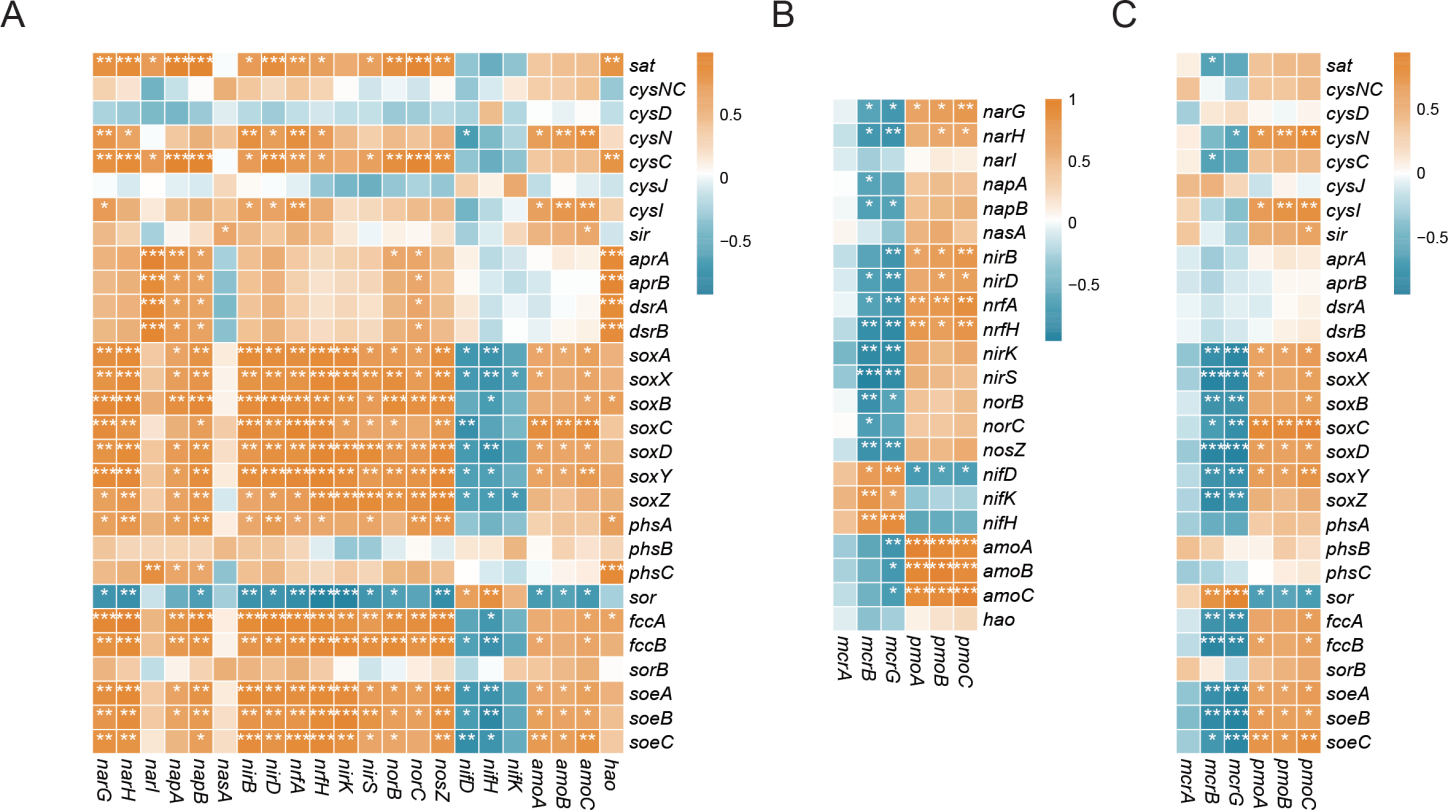
Heatmaps displaying Spearman's correlation coefficients between functional genes involved in nitrogen and sulphur (**A**), nitrogen and AOM/methanogenesis (**B**), and sulphur and AOM/methanogenesis (**C**) are presented. Correlation coefficients with *P* values less than 0.05, 0.01, and 0.001 are labeled with “*”, “**”, and “***”, respectively.

Sperman's correlation analysis was used to investigate the ecologically relevant relationships among methane, nitrogen, and sulphur cycling processes. Significant positive correlations (*P* < 0.05) were found between gene families related to sulphur oxidation (*sox*, *soeABC*, and *fccAB*), thiosulfate disproportionation (*phsA*) with denitrification (*narG*, *nirKS*, and *norB*C), and DNRA (*nirBD* and *nrfAH*), suggesting that denitrification and DNRA were coupled with sulphur oxidation and disproportionation (Fig. 3A). Furthermore, we observed significant (*P* < 0.05) and positive correlations between the *mcrBG* and *nifDHK* gene families, which implied a potential coupling between nitrogen fixation and methanogenesis/AOM (Fig. 3B). Regarding the relationship between methane and sulphur cycling processes, the abundance of *mcrBG* genes showed a positive correlation with the *sor* gene*,* suggesting that the sulphur disproportionation was likely to be coupled with AOM (Fig. 3C). In contrast, the study found negative correlations between the *mcrABG* and key genes specific to sulfate reduction such as *aprAB* and *dsrAB* (Fig. 3C). This result suggested that not all SRBs groups cooperated with the ANMEs to perform AOM.

### Genomic capability of coupling methane, nitrogen, and sulphur cycling processes

To better understand the metabolic capabilities of uncultured microbial species involved in methane, nitrogen, and sulphur cycling, we obtained partial and near-complete genomes to reconstruct the metabolic pathways of numerous community members. A total of 255 metagenome-assembled genomes (MAGs) (170 bacterial and 85 archaeal) were recovered with >50% completeness and <10% contamination (Table S2). These MAGs span nine archaeal and 29 bacterial phyla. Most of these MAGs had a relative abundance under 1% across all samples (Table S3). Archaeal genomes were strongly represented by Thermoproteota (mostly class Bathyarchaeia, *n* = 32), Thermoplasmatota (*n* = 15), and Halobacteriota (class Methanosarcinales and ANME-1, *n* = 11), while bacterial genomes were most abundant in Chloroflexota (mainly Dehalococcoidia) (*n* = 41) and Desulfobacterota (*n* = 29), WOR-3 (*n* = 15), Acidobacteriota (class Aminicenantia) (*n* = 13), and Aerophobota (formerly CD12) (*n* = 12) (Table S2). However, in our study, we were not able to obtain Atribacteria (JS1) MAGs, which showed the highest abundances by the analysis of 16S rRNA gene fragments in metagenomic libraries.

**Fig 4:**
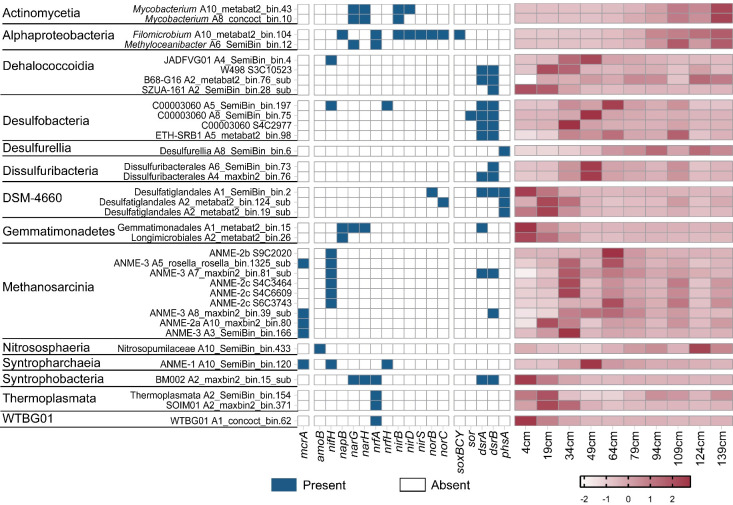
Presence of key genes involved in AOM/methanogenesis (*mcrA*), aerobic ammonia oxidation (*amoB*), nitrogen fixation (*nifH*), denitrification (*napB*, *narGH*, *nirS*, and *norBC*), DNRA (*nirBD* and *nrfAH*), sulfite oxidation (*soxBCY*), sulphur disproportionation (*sor*), dissimilatory sulfate reduction (*dsrAB*), and thiosulfate disproportionation (*phsAB*) across microbial MAGs (left). The chart on the right displayed the distribution of the functional MAGs mentioned above across the sediment depths. A detailed list of genes in the MAGs can be found in Table S4.

**Fig 5:**
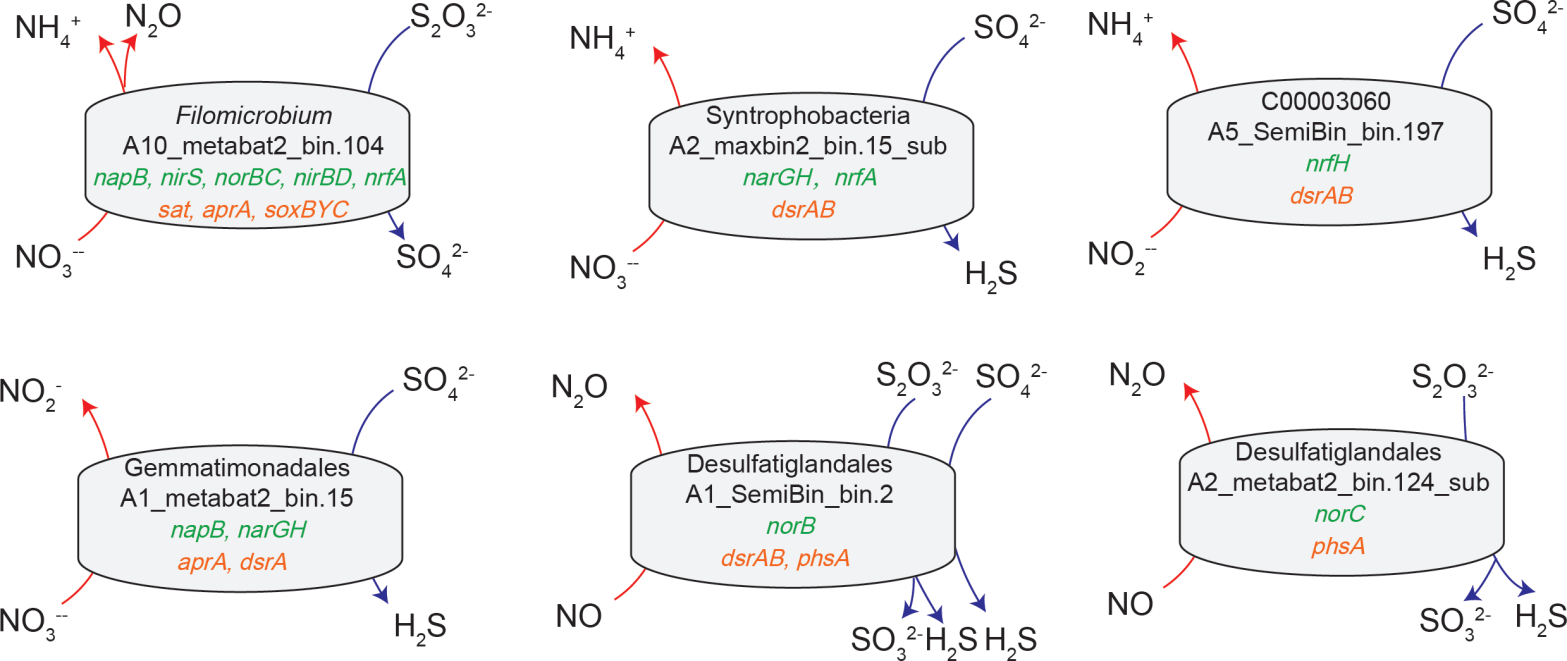
The metabolic models of the coupling of nitrogen and sulphur cycling processes in the six recovered MAGs.

The *nifH* gene, which is key for nitrogen fixation, was found in 10 MAGs. These MAGs belonged to SRB C00003060, ANMEs (ANME-1, ANM-2b, ANME-2c, and ANME-3), and heterotrophs Dehalococcoidia (Fig. 4). The metagenome analysis revealed a coupling between nitrogen and sulphur cycling processes in the OT metagenomes. One *Filomicrobium* MAG (A10_metabat2_bin.104) was particularly noteworthy due to its possession of partial genes involved in denitrification (*napB*, *nirS*, and *norBC*), DNRA (*nirBD* and *nrfA*), and sulphur oxidation (*soxBYC*) (Fig. 4 and 5). This MAG was more abundant in the deeper sediment (94–139 cmbsf) (Fig. 4). Two MAGs, Syntrophobacteria A2_maxbin2_bin.15_sub and C300003060 A5_SemiBin_bin.197, which were putatively identified as SRBs (Fig. 4 and 5), were found to harbor the key gene for DNRA (*nrfA* or *nrfH*) (Fig. 4 and 5). The Syntrophobacteria A2_maxbin2_bin.15_sub was most abundant in the surface sediment (4 cmbsf), while C300003060 A5_SemiBin_bin.197 was most abundant at 64 cmbsf (Fig. 4). In addition, one Gemmatimonadales MAG (A1_metabat2_bin.15) contained nitrate reductase genes (*narGH* and *napB*) as well as the key gene for sulfate reducing (*aprA* and *dsrA*) (Fig. 4 and 5). The A1_metabat2_bin.15 MAG had the highest relative abundance in the surface sediment (Fig. 4). Further examination revealed that two Desulfatiglandales MAGs (A1_SemiBin_bin.2 and A2_metabat2_bin.124_sub) contained key genes for thiosulfate disproportionation (*phsA*) as well as nitric oxide reductase genes (*norB* or *norC*) (Fig. 4 and 5）. Furthermore, the Desulfatiglandales A1_SemiBin_bin.2 also contained the sulfate reducing gene *dsrAB* (Fig. 4 and 5). These two Desulfatiglandales MAGs had their higher abundances in the above SMTZ (Fig. 4).

**Fig 6:**
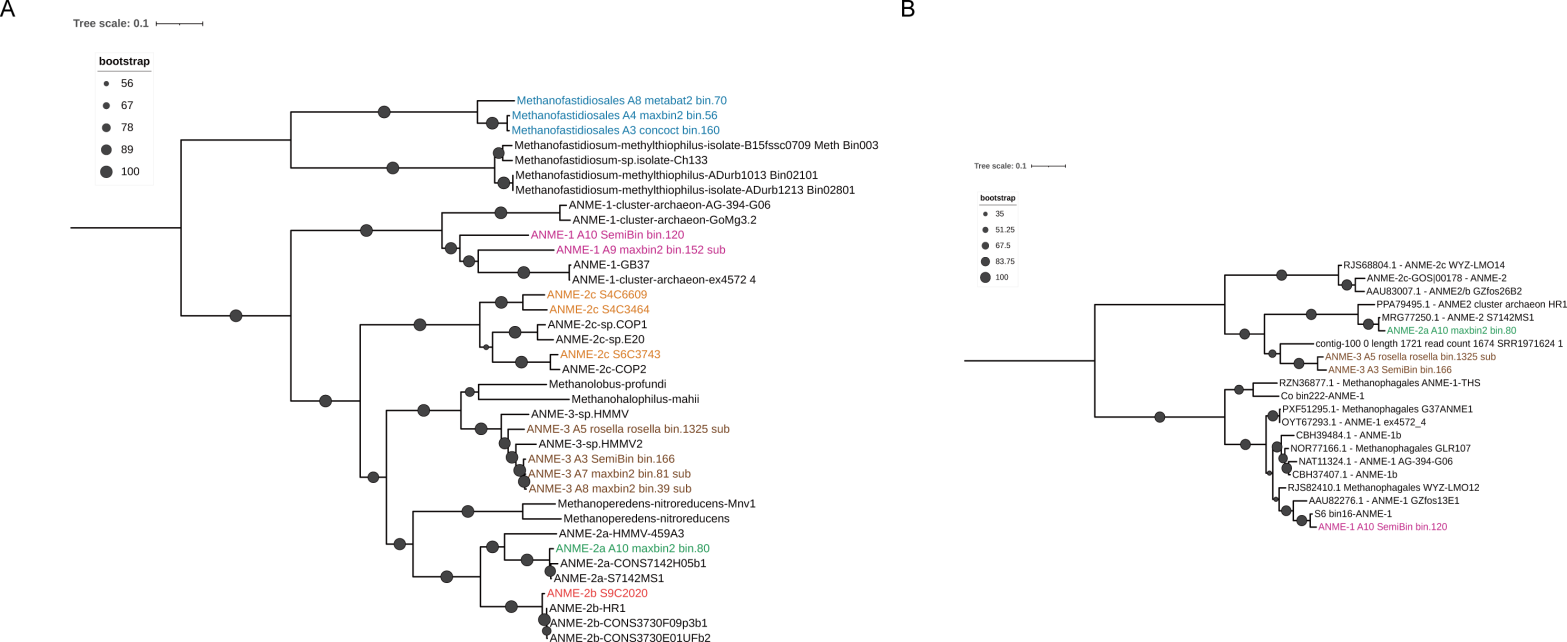
Maximum-likelihood phylogenetic trees of MAGs and the detected McrA sequences (**A**). Maximum-likelihood phylogenetic trees of MAGs based on alignments of 43 conserved protein sequences from the methanogen/ANME MAGs. (**B**) Phylogenetic tree constructed from alignments of amino acid sequences of *mcrA* genes. The MAG A8 maxbin2 bin.39 sub *mcrA* is not included in the tree because of its short length. Size of the solid dots represents the bootstrap value. The reference metagenomes for ANMEs and the reference sequences for *mcrA* were obtained from a previous study (40).

The *dsrAB* genes were also detected in MAGs within Dehalococcoidia (A2_metabat2_bin.76_sub, S3C10523 and A2_SemiBin_bin.28_sub), ANME-3 (A7_maxbin2_bin.81_sub and A8_maxbin2_bin.39_sub), and Gemmatimonadetes (A1_metabat2_bin.15), indicating their potential capability for sulfate respiration (Fig. 4 and Fig. S5). Three genomes of Desulfatiglandales (A1_SemiBin_bin.2, A2_metabat2_bin.124_sub and A2_metabat2_bin.19_sub) and one genome of Desulphurellia (A8_SemiBin_bin.6) were found to contain thiosulfate reductase genes (*phs*) (Fig. 4). These genes catalyzed the disproportionation of S_2_O_3_^2−^ to H_2_S and SO_3_^2-^. Additionally, the *sor* gene detected in one C00003060 MAG (A8_SemiBin_bin.75) suggested its possible involvement in sulfate oxidation, catalyzing S^0^ to SO_3_^2−^ and H_2_S (Fig. 4). Based on the rigorous classification using the GTDB and subsequent phylogenetic study, we classified a total of 14 MAGs as Methanofastidiosales, along with three clusters of anaerobic methanotrophs (ANME-1, ANME-2, and ANME-3) (Fig. 6A; Table S6). Unlike the historically studied Methanofastidiosales (39), our MAGs did not possess *mcr* and methyltransferase genes such as *mttB*, *mtbB*, and *mtmB*. The differences observed in our MAGs might be attributed to their incompleteness genomes, which ranged from 56.2% to 76.2%. Among the ANME MAGs, one ANME-1 MAG (A10_SemiBin_bin.120), one ANME-2a MAG (A10_maxbin2_bin.80), and three ANME-3 MAGs (A3_SemiBin_bin.166, A5_rosella_rosella_bin.1325_sub and A8_maxbin2_bin.39_sub) harbored *mcrA* genes (Fig. 6B). The abundances of the ANMEs MAGs were more abundant in the SMTZ sediments (below the 49 cm). The ANME MAGs also encoded the reverse of the CO_2_-dependent methanogenesis pathway (*fmd*/*fwd*, *ftr*, *mch*, *mtd*, *mer*, *mtr*, and *mcr*), which oxidizes methyl-CoM to CO_2_ (Fig. S4; Table S6).

**Fig 7:**
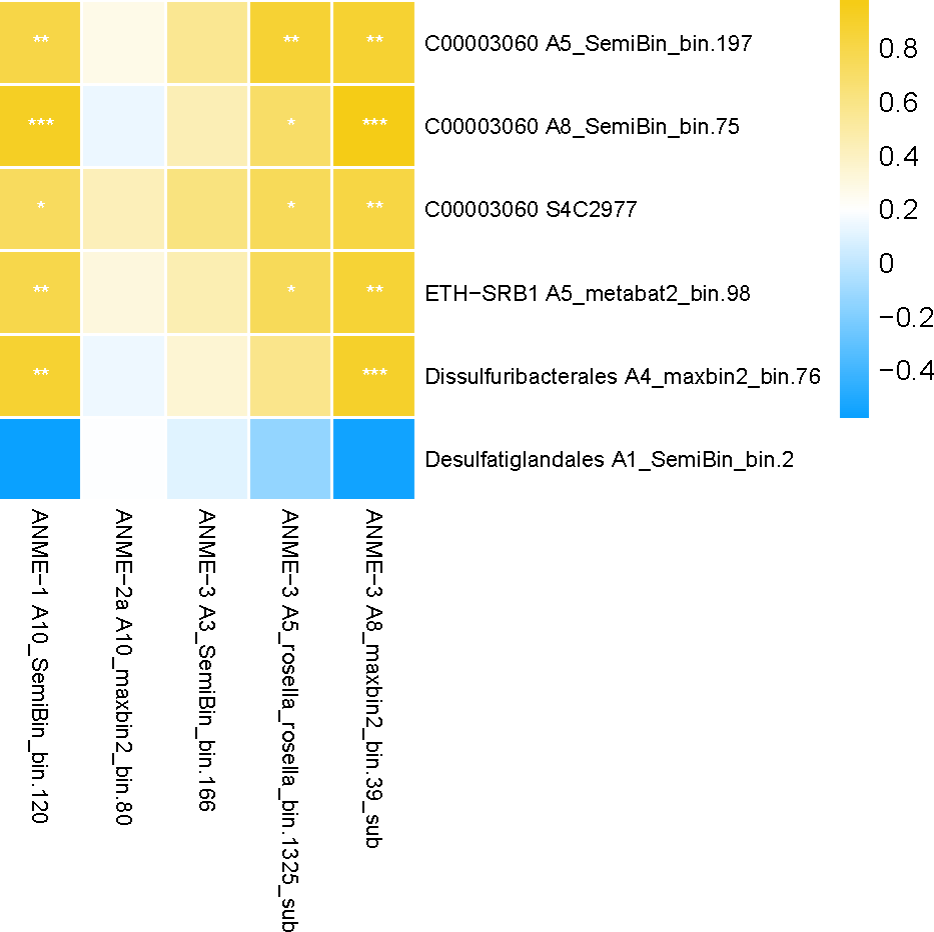
Heatmap of Spearman's correlation coefficients between the abundance of ANME MAGs and SRB MAGs. Correlation coefficients with *P* values less than 0.05, 0.01, and 0.001 are labeled with “*”, “**”, and “***”, respectively.

To gain deeper insights into the microbial consortia of ANMEs and SRBs in the G02, we analyzed the correlation of the abundance between the ANMEs MAGs harboring *mcrA* and Desulfobacterota MAGs harboring *dsrA* genes (Fig. 7). Our findings indicated that MAGs within ANME-1 and ANME-3 had a significant positive correlation with C00003060, Dissulphuribacterales, and ETH-SRB1 (*P* < 0.05) (Fig. 7). Furthermore, the ANME-1 and ANME-3 MAGs mentioned above contained genes for multiheme cytochromes, which are frequently involved in directly transferring electron to their SRB partners (Table S7). Taken together, these results suggested that SRBs within C00003060, Dissulphuribacterales, and ETH-SRB1 were syntrophic partners with ANME-1 and ANME-3. In contrast, the MAGs within Desulfatiglandales showed negative correlations with MAGs containing *mcrA*, suggesting that Desulfatiglandales were not the syntrophic partners of ANMEs.

### Dissimilatory iron reduction

Genes related to iron reduction were determined using FeGenie (Table S8). The multiheme cytochromes encoded by the DFE genes were detected in four SRB MAGs affiliated with order C00003060, ETH-SRB1, Dissulphuribacterales from the phylum Desulfobacterota. These multiheme cytochromes enable SRBs to directly utilize electrons from insoluble minerals in energy-poor marine sediments (41). Another study discovered that these multiheme cytochromes can perform extracellular electron transport or iron reduction in conjunction with other components of the flavin-based electron transport system (42). *OmcZ*, a gene encoding the outer surface c-type cytochrome involved in extracellular reduction of Fe (III) in *Geobacter sulphurreducens* biofilms (43), was identified in a MAG belonging to ANME-2b (Table S8 and Fig. S6). The category of iron reduction genes labeled as “other” includes those that encode hypothetical proteins attributed to porins and cytochromes. These genes were detected in one MAG affiliated with class MSB-5A5 (phylum Zixibacteria). Zixibacteria has been found to be capable of both oxidizing and reducing ferric/ferrous iron (44).

## DISCUSSION

In this study, we investigated the metabolic pathways for methane, nitrogen, and sulphur and their potential coupling mechanisms at a cold seep site in the OT. The study revealed that certain genes related to nitrification, denitrification, DNRA, sulphur oxidation, and thiosulfate disproportionation were more prevalent in the sediments above SMTZ, particularly in the surface sediment. Meanwhile, only genes related to sulphur disproportionation, methanogenesis/AOM, and nitrogen fixation were enriched in the sediments within SMTZ. Recent research suggested that the surface layer may act as a reservoir for microbial species and functions in deep-sea cold seep (45). Furthermore, the Spearman's analysis revealed complex microbial interactions in the OT cold seep sediments, including (i) sulphur oxidation coupled with dissimilatory nitrate reduction processes, such as denitrification and DNRA, and (ii) AOM coupled with sulphur disproportionation and nitrogen fixation. Then, we analyzed the metabolic pathways within microbial genomes in order to discuss their functional role in the biogeochemical cycles and the interactions between different biogeochemical cycles in the OT.

Microbial nitrogen cycling in the cold seep environments has received less attention compared to carbon and sulphur cycling. Cold seep sediments are highly rich in organic matter but limit in nitrogen for microbial growth. Diazotrophs play a critical role in alleviating nitrogen limitation in many marine ecosystems by fixing nitrogen. The key gene for nitrogen fixation, *nifH*, was mainly distributed in ANMEs MAGs. A recent study indicated that the AOM process can provide ATP for microbial nitrogen fixation (5). One *Filomicrobium* MAG appeared to be equipped to deal with a variety of processes, including denitrification, DNRA, and thiosulfate oxidation. The coupling of denitrification with the oxidation of reduced inorganic sulphur compounds has been detected in the cold seep sediments, which could be performed by autotrophic microbes such as Campylobacterota, Gammaproteobacteria, and Alphaproteobacteria (11, 46). Additionally, anaerobic oxidation of sulphur (47) and sulfide (48) coupled to DNRA has been observed in marine environments. Four MAGs containing putative SRBs (Syntrophobacteria, C00003060, Gemmatimonadales, and Desulfatiglandales) with *dsrA* genes also contained partial genes involved in denitrification and DNRA. The activity of either DNRA or denitrification for nitrate reduction appears to be a common feature among SRBs (49, 50), indicating a flexible metabolism depending on the availability of electron acceptors.

Most members of the SRB belonged to the phylum Desulfobacterota, including C00003060, ETH-SRB1, Dissulphuribacterales, and Desulfatiglandales (Fig. S5). Members of the order “C00003060” were corresponded to the lineage of SEEP-SRB1c (51). The close relatives of the order C00003060 are exclusively found in hydrocarbon-rich marine sediments, such as the Hydrate Ridge cold seeps off the Pacific coast (52) and GB hydrothermal sediments (53). SEEP-SRB1 has been shown to form coccoid consortia with ANME-1 (7). Dissulphuribacterales were identified as the dominant SRB groups in the SMTZ of the deep-sea cold seep sediments (11). In addition, members within ETH-SRB1 were proposed to form a syntrophic relationship with ethane-oxidizing archaea from ANME-2d (54). In this study, C00003060, Dissulphuribacterales, and ETH-SRB1 were identified as the syntrophic SRB partners with ANMEs. Desulfatiglandales were not found to be associated with ANMEs, but they were found to have the ability to disproportionate thiosulfate. In addition to Desulfobacterota, Dehalococcoidia, ANME-3, and Gemmatimonadetes have also demonstrated their ability to respire sulfate. Members of Dehalococcoidia and Gemmatimonadetes have been shown to reduce sulfate (55, 56). The Dsr in ANME-3 MAGs was intriguing (Fig. 4). ANME-3 appeared to exist in marine methane seeps where methane was vigorously emitted, such as the methane hydrate at Hydrate Ridge (57), the Sonora Margin cold seeps (58), and the Haakon Mosby Mud Volcano (59). ANME-3 members were detected both in near-surface sediments with comparatively higher sulfate concentrations (58, 59) and in sediments within and below the SMTZ (60). Our data suggest that the ANME-3 members may be capable of performing the AOM process independently, providing valuable insight into the metabolic strategy of ANME-3 in the cold seep sediments. However, it should be noted that there was a possibility of a binning error or incorrect annotation for the ANME-3 genomes. Further research is required to confirm the accuracy of the data.

In the present study, Methanomassiliicoccales and Methanofastidiosales were detected as the dominant methanogens in the OT cold seep sediments. These methanogen members are commonly found in methane hydrate-associated marine sediments, such as sediments from the Shenhu area and Qiongdongnan Basin of the South China Sea（^56^, ^61^), eastern Nankai Trough, and sediments below the SMTZ from the Shimokita Peninsula of Japan (62). The OT sediments may undergo methylotrophic methanogenesis, as Methanofastidiosa and Methanomassiliicoccales have been reported to perform this process by reducing methanol and methylamines while oxidizing hydrogen and reducing inorganic carbon (39). Methylotrophic methanogenesis has been shown to be important in subseafloor anaerobic sediments (63). Although, SRBs dominated the cold seep sediments, outcompeting methanogens for common substances such as hydrogen or acetate, this competition can be relieved through the use of noncompetitive C1 substrates by methylotrophic methanogens (64–66).

The Okinawa Trough (OT) is characterized by the coexistence of cold seeps and hydrothermal activities, particularly in the middle OT (18, 67). Hydrothermal Fe can be transported over long distances from the hydrothermal plumes in the open ocean (68, 69). The G02 core is located approximately 45 km away from the Minami-Ensei Knoll (MEK) hydrothermal field and 150 km away from other hydrothermal fields (68, 69). It has been estimated that the site received a large amount of hydrothermal materials from the MEK hydrothermal field, which is driven by the Kuroshio Current (23). The relative contribution of hydrothermal Fe fractions in the G02 was estimated to be 16.8% (23). In anaerobic marine sediments, iron oxides can undergo abiotic reactions with hydrogen sulfide leading to the formation of pyrite. OT cold sediments in the SMTZ have been shown to have a high degree of pyritization, which facilitated by SR-AOM (23). During the formation process of pyrite, intermediate sulphur species such as thiosulfate (S_2_O_3_^2−^), sulfite (SO_3_^2−^), tetrathionate (S_4_O_6_^2−^), and elemental sulphur (S^0^) were produced (70). These reduced sulphur species, which are disproportionate to hydrogen sulphide and sulphate, would act as an electron acceptor for AOM. The study found a significant positive correlation between *sor* and *mcrBG* (Fig. 3), indicating a coupling relationship between AOM and sulphur disproportionation. One SRB C00003060 MAG (A8_SemiBin_bin.75), which contained the key gene involved in sulphur disproportionation (*sor*), was found to be associated with MAGs of ANME-1 and ANME-3 (Fig. 7). Therefore, it was possible that SRB C00003060 was involved in a syntrophic relationship with ANMEs, potentially linking the cycles of sulphur disproportionation and AOM in the OT cold seep sediments. AOM has been detected in association with sulphur disproportionation driven by zerovalent sulphur in the deep terrestrial subsurface (71). An exciting aspect of the ANME-2b genome was that it contained a gene (*omcZ*) that could be pivotal for electron transfer, implying a capability for iron-coupled AOM. The geochemical evidence from the seep carbonates suggested that iron reduction coupled to AOM may have ever been prevalent at the site G02 (22). However, further studies should focus on enhancing and characterizing the physiology of ANME-2b to improve our understanding of its biological function *in situ*.

### Conclusion

Analysis of the metagenome sequencing data revealed the microbiomes responsible for the vertically stratified cycling of methane, nitrogen, and sulphur in the sediments of the OT cold seep and their coupling mechanisms. Most pathways involved in nitrogen and sulphur cycling were enriched in the surface sediment. Based on the correlation between functional genes, it could be inferred that sulphur oxidation with denitrification and DNRA, AOM with sulphur disproportionation, and nitrogen fixation occurred. This hypothesis was confirmed by the subsequent results of the study. The potential role of the *Filomicrobium* member in coupling denitrification and DNRA with sulphur oxidation was highlighted. The key gene for nitrogen fixation was mainly distributed in MAGs of ANMEs. The SRB C00003060 contained key genes related to sulphur disproportionation (*sor*), which were found to be associated with ANME-1. Additionally, the study revealed that, apart from Deltaproteobacteria, bacterial members such as Dehalococcoidia and Gemmatimonadetes were predicted to be capable of sulfate reduction. The ANME-3 genome contained key genes for both sulfate reduction (*dsr*) and methane oxidation (*mcr*), indicating that this member can mediate AOM independently. In the same MAG, Syntrophobacteria, Desulfobacteria, and Gemmatimonadales underwent simultaneous sulfate and nitrate/nitrite reduction. Our observations have highlighted H_2_-dependent methylotrophic methanogens, specifically Methanomassiliicoccales and Methanofastidiosales, as the primary methanogenic group. These findings improve our understanding of the microbial processes driving nutrient cycling in the OT cold seep environment.

## Data Availability

All metagenomic raw reads used in this study are available at the Sequence Read Archive under BioProject accession number PRJNA1014595. All MAGs used in this study have been deposited in can be found in figshare (https://figshare.com/articles/dataset/_/24742845).

## SUPPLEMENTAL MATERIAL

Supplemental Figures spectrum.03490-23-s0001.docx Fig. S1-S6.

Supplemental Tables spectrum.03490-23-s0002.xlsx Tables S1-S8.

ASM does not own the copyrights to Supplemental Material that may be linked to, or accessed through, an article. The authors have granted ASM a non-exclusive, world-wide license to publish the Supplemental Material files. Please contact the corresponding author directly for reuse.
