# Supplementary material for: Potential coupling of microbial methane, nitrogen, and sulphur cycling in the Okinawa Trough cold seep sediments
Source: Microbiol Spectr. 2024 May 1;12(6):e03490-23. doi: 10.1128/spectrum.03490-23 (PMC11237511; doi:10.1128/spectrum.03490-23)
Supplement: Supplemental Figures — Fig. S1-S6. [file spectrum.03490-23-s0001.docx]

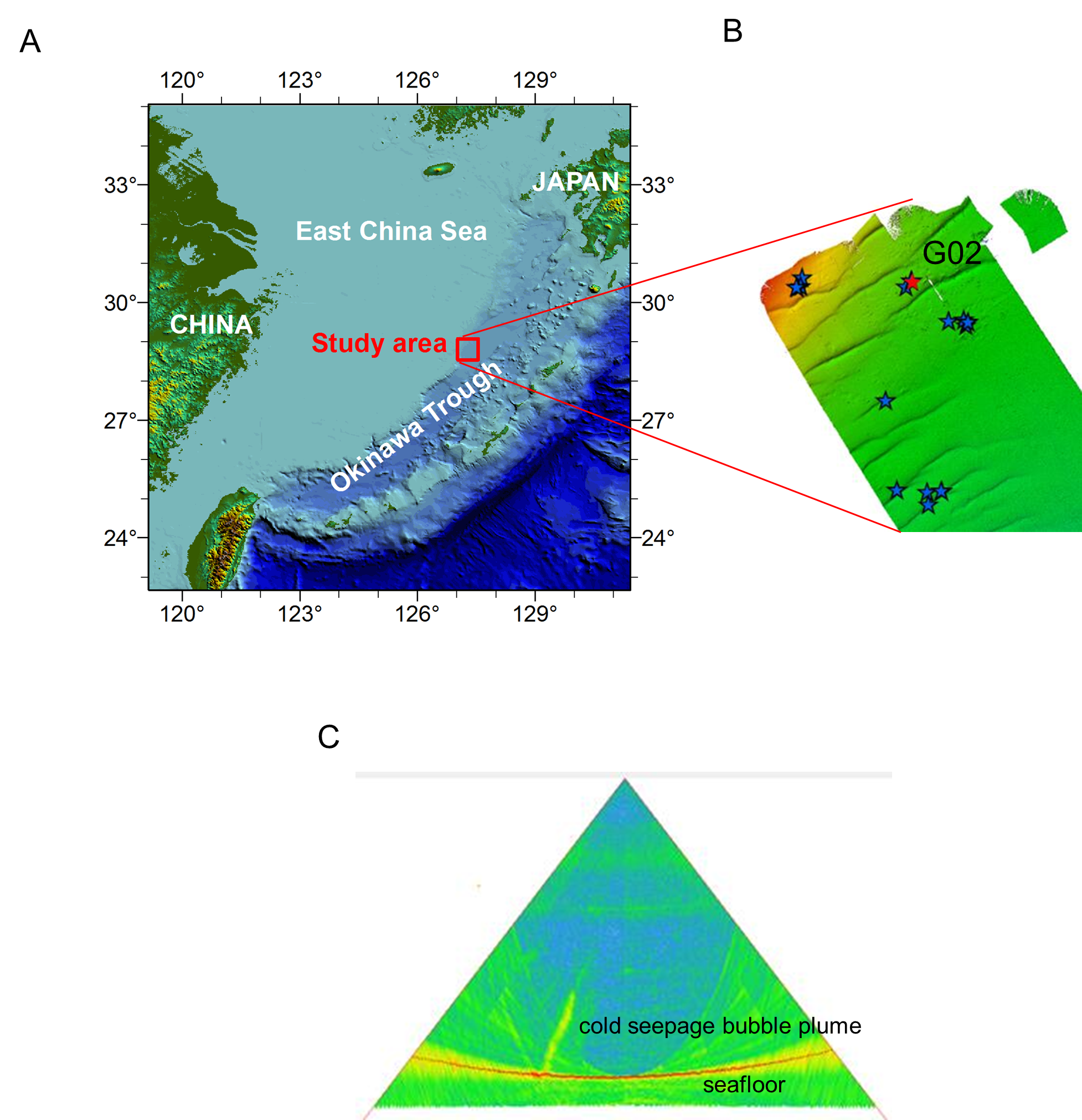


**Fig. S1** Topographical profile of the OT (A), multibeam topographic map (B) and multibeam backscatter image (C) of the study area. (In the multibeam topographic map (B), the five-pointed stars represent the distribution of acoustic plumes in water, and the red five-pointed stars represent sampling site G02). (1)


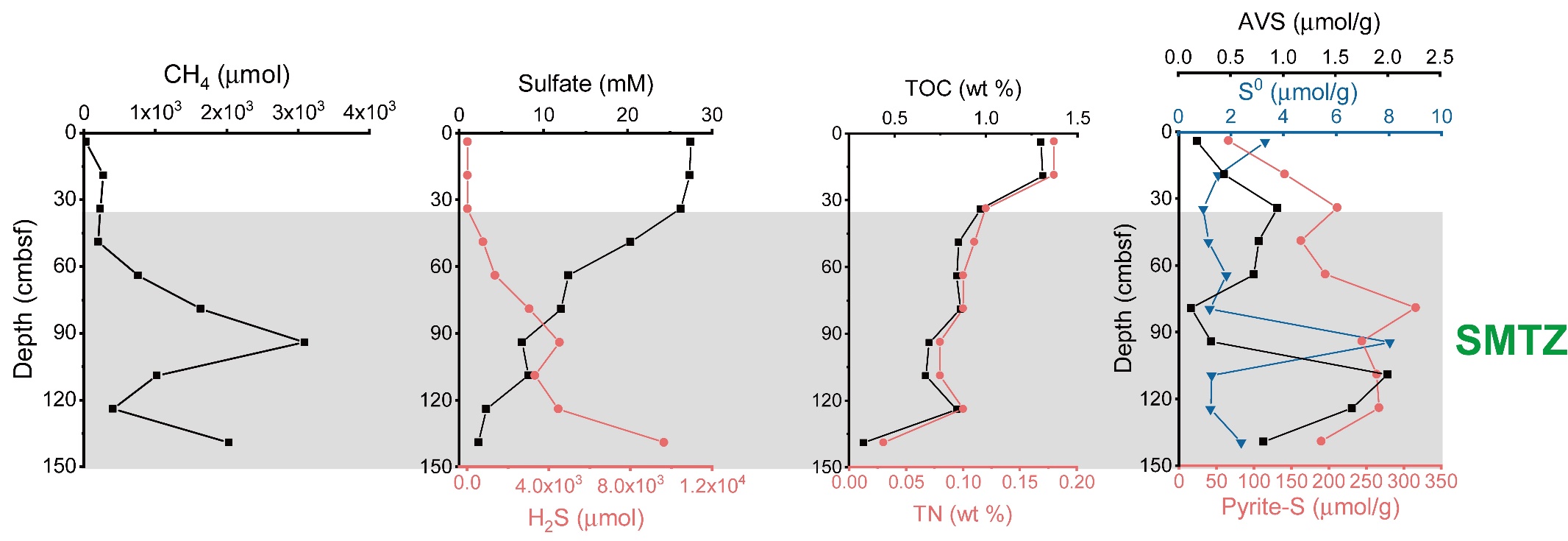


**Fig. S2** Vertical profiles of the CH_4_, sulfate, H_2_S, TOC, TN, acid volatile sulfide (AVS), elemental sulfur (S^0^), pyrite-S (S_py_) in the G02 core. (redrawn from the previously studies(1, 2)).


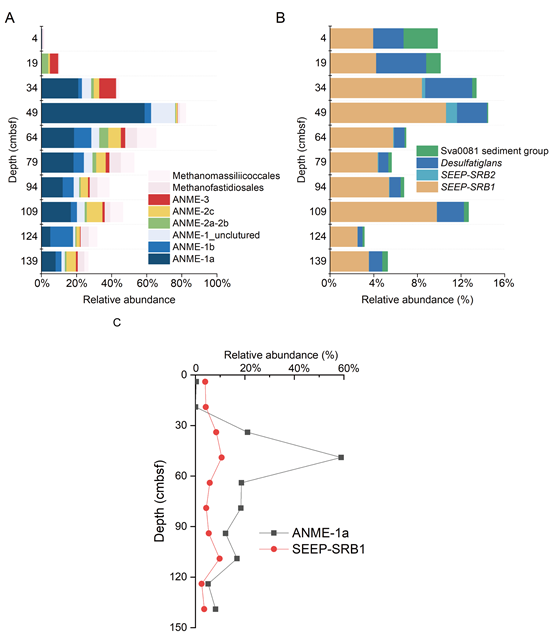


**Fig. S3** Compositions of anaerobic methanotrophic (ANME)/methanogens (A), sulfate reducing bacteria (SRB; B) and profiles showing the relative abundance of ANME-1a and SEEP-SRB1.


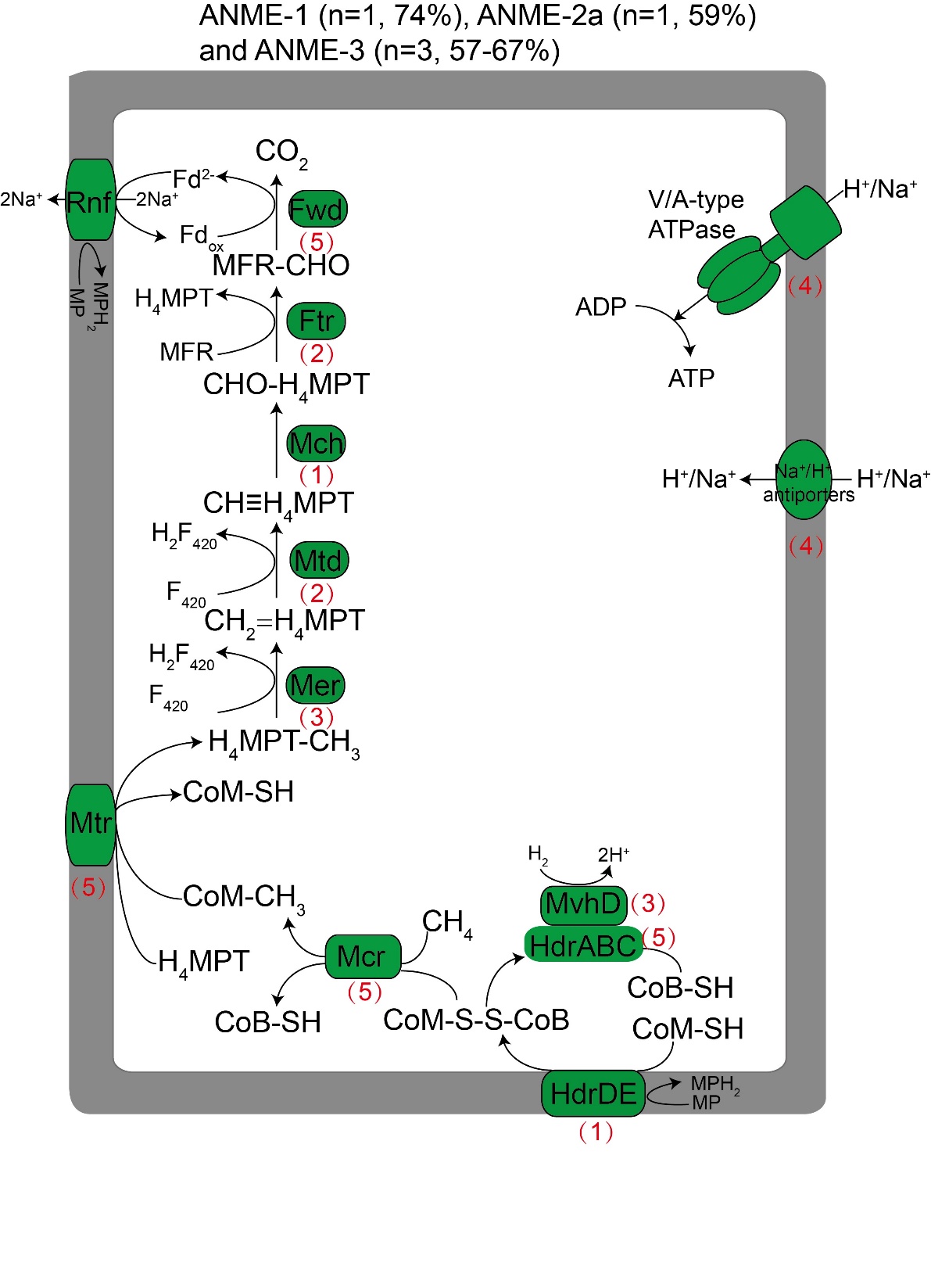


**Fig. S4** Predicted metabolic models for anaerobic oxidation of methane based on archaeal MAGs with *mcrA* genes. The red font indicates that not all of the retrieved MAGs encode the enzyme and the number of MAGs encoding each enzyme are showed in parentheses.


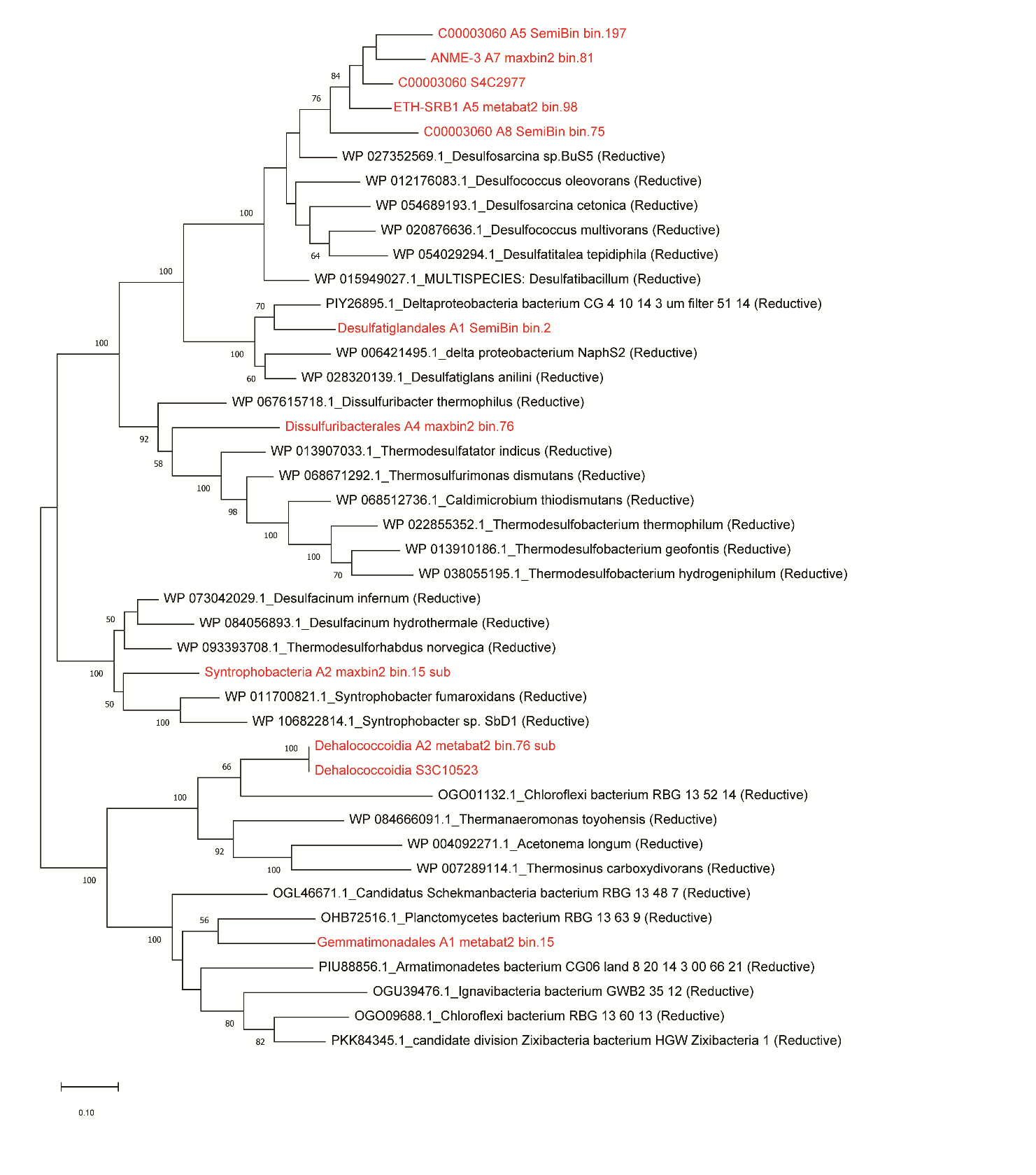


**Fig. S5** Maximum-likelihood tree of the amino acid sequences of the dissimilatory sulfite reductase A subunit (DsrA). The tree was generated using the JTT matrix-based model by MEGA11 from 50 bootstraps and midpoint-rooted. Sequences from the cold seep metagenome-assembled genomes obtained in this study are shown in red. The reference sequences for *dsrA* were obtained from a previous study (3).


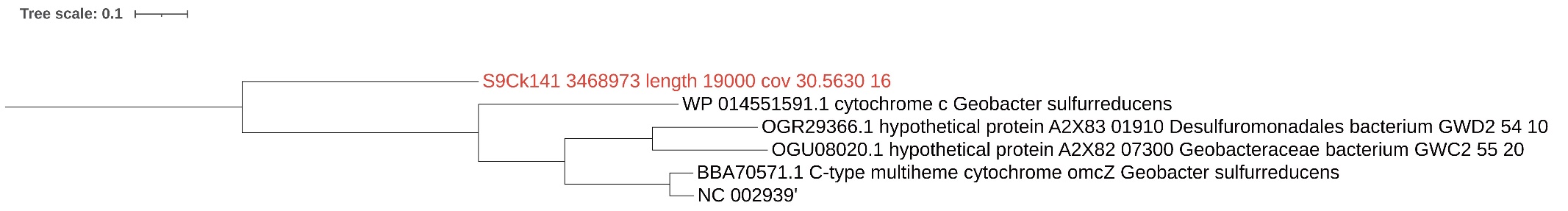


**Fig. S6** Maximum-likelihood tree of the amino acid sequences of the c-type cytochrome OmcZ. The tree was generated using the JTT matrix-based model by MEGA11 from 50 bootstraps and midpoint-rooted. Sequences from the cold seep metagenome-assembled genomes obtained in this study are shown in red. The reference sequences for *omcZ* were obtained from a previous study (4).

Reference

1. Chen Y, Xu C, Wu N, Sun Z, Liu C, Zhen Y, Xin Y, Zhang X, Geng W, Cao H, Zhai B, Li J, Qin S, Zhou Y. 2022. Diversity of Anaerobic Methane Oxidizers in the Cold Seep Sediments of the Okinawa Trough. Frontiers in Microbiology 13.

2. Qin S-S, Zhu M-X, Sun Z, Li T, Zhang X, Geng W, Cao H, Xu C, Zhai B, Chen Y. 2023. Diagenetic Geochemistry of Iron, Sulfur, and Molybdenum in Sediments of the Middle Okinawa Trough Impacted by Hydrothermal Plumes and/or Cold Seeps. Earth and Space Science 10:e2022EA002709.

3. Pok Man L, Chris G. 2020. Dissimilatory sulfite reductase DsrA sequences.fasta doi:10.26180/13152632.v1.

4. Garber AI, Nealson KH, Okamoto A, McAllister SM, Chan CS, Barco RA, Merino N. 2020. FeGenie: A Comprehensive Tool for the Identification of Iron Genes and Iron Gene Neighborhoods in Genome and Metagenome Assemblies. Front Microbiol 11:37.
